# Pedagogical Orientations and Evolving Responsibilities of Technological Universities: A Literature Review of the History of Engineering Education

**DOI:** 10.1007/s11948-023-00460-2

**Published:** 2023-12-05

**Authors:** Diana Adela Martin, Gunter Bombaerts, Maja Horst, Kyriaki Papageorgiou, Gianluigi Viscusi

**Affiliations:** 1 Philosophy and Ethics, Industrial Engineering and Innovation Sciences, Eindhoven University of Technology, Eindhoven, The Netherlands; 2 Faculty of Arts, Aarhus University, Aarhus C, Denmark; 3 Fusion Point, Esade Business and Law School, Barcelona, Spain; 4 Business School, Imperial College London, London, UK; 5 Centre for Engineering Education, University College London, London, UK; 6 Department of Management and Engineering, Information Systems and Digitalization, Linköping University, Linköping, Sweden; 7 Department of Interdisciplinary Studies of Culture, Norwegian University of Science and Technology, Trondheim, Norway

**Keywords:** History of engineering education, Institutional responsibility, Responsibility of technological universities, Societal responsibility, Engineering ethics education, Literature review, Values of technological universities

## Abstract

Current societal changes and challenges demand a broader role of technological universities, thus opening the question of how their role evolved over time and how to frame their current responsibility. In response to urgent calls for debating and redefining the identity of contemporary technological universities, this paper has two aims. The first aim is to identify the key characteristics and orientations marking the development of technological universities, as recorded in the history of engineering education. The second aim is to articulate the responsibility of contemporary technological universities given their different orientations and characteristics. For this, we first provide a non-systematic literature review of the key pedagogical orientations of technological universities, grounded in the history of engineering education. The five major orientations of technological universities presented in the paper are technical, economic, social, political, and ecological. We then use this historical survey to articulate the responsibilities of contemporary technological universities reflecting the different orientations. Technological universities can promote and foster the development of scientific, professional, civic, legal, or intra- and inter- generational responsibility. We argue that responsibility is not specific to any particular orientation, such that the concept is broadened to complement each orientation or mix of orientations of a technological university. Our contribution thus serves as a call for technological universities to self-reflect on their mission and identity, by offering a lens for identifying the orientations they currently foster and making explicit the responsibility arising from their current orientation or the ones they strive to cultivate.

## Introduction

Contemporary technological universities (TUs) are heterogenous in terms of their breadth and scope (Geschwind & Broström, 2020, p.15; Jamison et al., 2014; Edström, 2018). Their missions changed and broadened over time, either in response to the needs and demands of external stakeholders, to enhance their status or due to technological developments (Lecuyer, 1992; Seely, 1984). Edström (2017a, 2018, 2020) highlights the “dual nature” of engineering education, split between its academic function of purveying theoretical scientific knowledge and its professional function, oriented towards industry and practice. The two approaches are contrasted as representatives of the “shop culture” versus “school culture” (Seely, 1999a). In their seminal paper, Jamison, Kolmos & Holgaard (2014) mention a third historical orientation of engineering education emphasizing social service.

The engineering profession is also heterogeneous. Despite a common founding in science and mathematics, there is little unity between different specializations and a diverse social division of labor where “engineers work in different areas and apply their knowledge to diverse needs” (Sjöstrand, 2013, p.1; Larson, 2006; Meiksins & Smith, 1996; Case, 2017). Furthermore, Harwood (2006, p.53) acknowledges a “Janus-faced nature of engineering as an activity” due to its double binding ties with the two “worlds of science and of practice,” as evidenced in Germany (Gispen, 1989), the USA (McMahon, 1984) or Colombia (Valderrama et al., 2009).

As such, it appears that “engineering is not one profession but many” (Larson, 2006, p.541). The same can be said about engineering education, as it encompasses varied approaches. The heterogeneity of engineering education led to persistent tensions and challenges for TUs (Geschwind & Broström, 2020, p.15), resembling the “swinging movement of a pendulum as first practice and then theory prevailed” (Seely, 1999a, p.42; 2005). The challenge of the harmonious integration of varied educational approaches “remained remarkably consistent over time” (Seely, 2005, p.125). The existing tension was further exacerbated by “a general crisis” of engineering education, which called for “a new engineer” pursuing social responsibility (Beder, 1998; Christensen et al., 2007, pp.13–4; Conlon, 2008).

Our contribution responds to growing calls for recasting the discourse surrounding engineering and the conceptualization of engineering education (Froyd, 2011; Graham, 2012; van den Hoven, 2019). There is a need to provide an appropriate normative conception of the modern university that identifies its aims and values, thus providing direction to institutional designers (Miller (2019, p.1680). These calls are of increasing importance given the magnitude of current societal changes and challenges, which demand a broader role of TUs. They leave open the question of how the role of TUs evolved over time and how to frame their current responsibility.

In response to the need for debating and redefining the identity of contemporary technological universities, the paper aims to understand the development of TUs by looking back at the history of engineering education to identify their key characteristics and orientations. We then use this historical survey to look forward toward the prospects of TUs to promote and foster the development of responsibility. Our stance is that any orientation or mix of orientations of a TU comes with specific responsibilities. For this, we articulate a range of responsibilities for contemporary TUs, in alignment with each orientation. Thus, the research questions addressed in this paper are:

- RQ 1: What are the key characteristics and orientations marking the development of TUs, as recorded in the history of engineering education?
- RQ 2: What are the responsibilities of contemporary TUs given the different characteristics and orientations recorded in the history of engineering education?

## The Technological University: Between Its Theoretical Construct and Empirical Instantiation

Before analysing the main features and instantiations of TUs in the history of engineering education, a conceptual clarification is needed to elucidate the legitimacy of discussing TUs as distinct higher education institutions. This can be traced to specific practices, routines, and rituals noted in history (Larsen et al., 2020), on similar grounds to how business and medicine are considered to distinguish themselves as specialized higher education institutions (Augier & March, 2011; Flexner, 2002). The article does not aim to provide a definite answer about the definition and demarcation criteria for a TU. The focus lies on identity formation based on patterns about the ideas (and ideals) associated with the instantiations of distinct orientations recorded in the history of engineering education.

For this, we look at the institutional level to understand the different ways of conceiving the historical instantiations of TUs (or their counterparts^1^). We assume a distinction between the major concepts in use and their empirical instantiations, allowing for ideations of the TU and its institutional capability to develop towards these idealized directions. In this sense, we focus on the TU both as an organization and as an “assemblage of ideas” (Barnett, 2012, p.44).

When referring to the instantiated TU, we consider it an organizational entity that has identity and agency.^2^ We assume that a TU has identity inasmuch as it can be associated with a set of characteristics, articulated in programmatic documents, vision plans, mission statements or university mottos. The organizational identity of the TU can be seen as the outcome of a joint process of self-observation, by which its operations are reported through financial figures or annual reports, and self-description, in the form of an explicit message created collaboratively by its members to describe in general terms its operations (Seidl, 2005). As such, we encounter self-articulated identities of TUs such as MIT (2020) which is “striving for the highest standards of intellectual and creative excellence,” NMITE (n.d.) as “a (politely) disruptive game changer,” or TU Eindhoven (2018, p.13) described as “open, personal and engaged”. Considering this, the first research question of the contribution can be understood as facilitating an institutional identity-seeking and sense-making process rooted in historical evidence.

We also assume that a TU has agency inasmuch as such institutions have “options before them as to which direction to follow, which values to uphold and within which frameworks they might comport themselves” (Barnett, 2021, p.274). For example, Colorado School of Mines (n.d.) strives for its “ideas, actions and innovations to have *a transformative impact on individuals and society,* leading to shared prosperity and sustainable use of the Earth’s resources”. University College London (n.d.) "believes in contributing to local communities". The College of Engineering and Applied Science of the University of Cincinnati (n.d.) describes itself as a place where students “gain real-world experience while collecting a real-world paycheck.” In terms of values, TU Munich prioritizes excellence, entrepreneurial mindsets, integrity, collegiality, and resilience. Cape Peninsula University of Technology names as its first values those of “dealing with others in a spirit of Ubuntu” and “mutual respect*.”* Given this agential capacity, we can reprise the question asked by Terrance (1991, p.150) of “how are we to think about the moral responsibility of institutions?” in the context of TUs. This concern is reflected by our second research question, which aligns with Terrance (1991, p.160)’s belief that “the manner in which universities perform their responsibilities is an important component of our collective moral life” and bears “a significant influence on the moral understanding and behaviour of others.”

## Methodology

Our novel contribution is to frame the responsibility of TUs based on a historical analysis of implicit and explicit concepts, visions and pedagogical approaches documented in engineering education. The analysis is based on a non-systematic literature review using the methodological recommendations developed by Borrego et al. (2014). For this, we first set the research questions for the study and the criteria for selecting relevant studies. The inclusion criteria considered whether the documents discussed key moments in the development of engineering education, as well as aspects related to engineering education policymaking, implementation or teaching. Afterwards, we searched for relevant publications in the SCOPUS and Web of Science databases, using the following search string: TITLE-ABS-KEY (histor* AND engineering AND education). The sources retrieved via this search process were screened for relevance to the research questions stated above. In addition, the bibliographies of the most cited publications were screened to identify any other relevant publications. We also considered the literature consulted by the first author in her doctoral study (Martin, 2020) to include any further relevant sources. As such, the review included for full text screening more than 700 sources published in science and technology studies and engineering education journals, book chapters, the proceedings of major conferences and policy documents.

The search was conducted in English, and solely sources published in English were considered. Thus, a limitation is the framing of the concept of responsibility and the deployment of a historical lens provided by sources of an Anglo-speaking and Western-culture provenance. This limitation suggests the need to complement this study with commentaries or additional review studies set in other cultural and geographical contexts that would expand our proposal.

## The Historical Orientations of Engineering Education

In what follows, we present the major orientations encountered in the history of engineering education, exploring their emergence, the practices by which they are transposed in TUs, and the main criticisms. All these orientations are synthesized in Table 1. We follow Barnett (2011) to posit that TUs may have multiple orientations and that no institution instantiates solely one orientation. These orientations pertain to the TU’s academic function and the professional identity, values, and sense of responsibility fostered.

**Table 1:** Historical orientations of technological universities in engineering education (expanding Jamison, Kolmos & Holgaard, 2014)

	Science	Market	Social	Policy	Ecology
Conception of engineering	(Applied) science and technology	Economic and Technological innovation	Societal wellbeing	Public service	Environmental and Sustainable practice
Role of the engineer	Expert Consultant, Theoretician	Entrepreneur Manager	Community member	Legislator Regulator Policy-maker	Planetary citizen Transition manager Change agent
Function	Academic and research	Commercial and Entrepreneurial	Communitarian	Political	Global and planetary
Pedagogic focus	By the book, Theoretical MOOC	Practical Design- and project-based learning Lifecycle development CDIO principles	Contextual Science and Technology Studies Liberal Arts Communities of practice	Envisioning and future-casting (back- and fore-casting) Rhetorical	Learning by doing and doing by learning Systems thinking Humanitarian engineering
Values and engagement in value-creation	(Scientific) discovery (Research) integrity Excellence Disciplinary knowledge Expertize Objectivity Open science Academic freedom	(Product and process) innovation Scalability Efficiency Effectiveness, Competitiveness Profit Productivity	Justice Inclusiveness Care Equity Accessibility Subjectivity Empowerment	(Legal) accountability Public engagement Leadership Power (top-down)	Principles of sustainable development SDG Universality Non-discrimination
Stakeholders in value co-creation	R&D centers Laboratories	Start-ups SMEs Large industries	NGOs Citizen Associations Local Communities Consumer and user groups	Local and national governments, Think-tanks, National or international policy bodies	Environmental associations, groups and networks Humanitarian bodies Underprivileged or at-risk communities Communities affected by natural or man-made disasters
Responsibility	Scientific (per ethical review boards)	Professional (per professional codes of conduct and corporate social responsibility)	Civic (towards people and communities as ends in themselves)	Legal (per normative legal frameworks and regulations)	Intra-generational Inter-generational (per indirect reciprocity and ageless duties)

### The Scientific Orientation

The scientific orientation has a long tradition, first brought to the forefront of engineering education at the École Polytechnique in Paris during the French Revolution. It was further adopted and adapted throughout the industrialized world during the nineteenth and twentieth centuries (Angulo, 2012; Jamison, 2013), counting among its representatives at their origins the Danish Technical University, the Czech Polytechnic University of Prague, Karlsruhe Institute of Technology or the Technical University of Vienna (Jessen, 2007; Bucciarelli et al., 2009; Jamison, 2013; Geschwind & Broström, 2020)^3^. According to its first prospectus published in 1826, the Civil Engineering Degree of University College London (n.d.) aimed to offer a “system of academical education” to the “young men intended for the scientific profession of Civil Engineer”.

This orientation fosters an academic vision of engineering as applied science, which emphasizes the imparting of scientific knowledge through educational processes consisting largely of “book learning,” lectures, small group discussions to support students struggling with the theoretical material, and practical sessions about machine drawing and applying the principles of mathematics and physics to the construction of roads, waterways, military structures or weaponry (Angulo, 2012 p.318; Jamison, 2013, p.21; Fox & Guagnini, 2004).

The science-driven curriculum favours mathematics and the natural and physical sciences of specific engineering fields. In this sense, “the norms and values are often focused on academic freedom, and power and influence is largely rooted in disciplinary knowledge and expertise” (Pinheiro & Stensaker, 2014, p.500). This educational approach prepares engineers for the roles of experts, consultants, or theoreticians (Hashagen, in Harwood, 2006; Jamison et al., 2014; McMahon, 1984). According to Jamison (2013, p.21), the professional identity is of an applied scientist who “transfers theories and concepts of the basic sciences into one or another instrumental materialization”. Such an engineer is a “no-nonsense problem solver, guided by scientific rationality” (Herkert, 2001).

The scientific orientation had a prominent role in the history of engineering education (Petrina, 2003), and is still considered to dominate it (Martin et al., 2021a). According to Wicklein (1997, p.72), the engineering curriculum is “little different from the old vocational models used in years past that concentrate on the technical aspects of selected tools and materials”.

Nevertheless, in recent decades, this orientation was criticized for its commitment to value neutrality and the decoupling of the scientific discourse from societal concerns and public welfare (Carberry & Baker, 2018; Cech, 2014; Martin & Polmear, 2023). More so, it is argued that TUs’ exclusive focus on a scientific orientation does “an insufficient job in giving students the experience of engineering as a meaningful craft, or that as engineers they will be able to contribute to a better world” (Stevens et al., 2007, p.3). In its extreme form, the disengagement from societal problems of the scientific orientation is predicated to give rise to what Barry (2012) describes as “technically competent barbarians,” a coinage referring to the engineers developing technological solutions that move humanity on an unsustainable path.

### The Market Orientation

Since the beginning of engineering higher education, the market orientation was cast in opposition to the scientific one (Edström, 2017a, 2020; Seely, 1999a; Harwood, 2006). In many European regions, the rationale behind the development of TUs was linked with tackling industrial decline or boosting economic competitiveness (Ahlström, 1982; Geschwind & Broström, 2020). This is the case of The Netherlands, where the founding of the Technical University of Twente in 1961 is considered “a particularly clear example of how national and regional level strategies to facilitate a transition from failing industrial structures to a new regime of technology-based entrepreneurship were strongly anchored in ideas about what a technical university would be able to achieve in this domain” (Geschwind & Broström, 2020, p.20). In Belgium, Liège was chosen by Royal decree to the detriment of Namur as the site for providing technical education due to its proximity to coalmines, ironworks, and quarries. As such, the University of Liège incorporated a Mining School in 1825. Here, the University’s administrator promoted a different vision to the one of the Paris Polytechnic model by introducing practical training and fieldwork. This served to develop links with local industries, including courses held in John Cockerill's enterprise at Seraing, which was one of the major and most modern ironworks of the time (Leboutte, 1995, pp.102–103). The situation was similar in Sweden, where technological education initially reflected the profile of local industries: “education in mining was provided in the mining town of Falun, shipping and textiles in the port city of Gothenburg, and machine engineering and chemical technology in the capital Stockholm” (Geschwind & Broström, 2020, p.16).

In England and Wales, technical education began to develop at a time when Great Britain’s economic strength was challenged by Germany’s rising power, thus emphasizing industrial relevance (Walsh, 2018). It came into force with the signing of the Technical Instruction Act in 1889, which promoted “instruction in the principles of science and art applicable to industries, and in the application of special branches of science and art to specific industries or employments” (Commission on Technical Education, p.33, in Walsh, 2018). As Heywood (2005, p.5) notes, in Great Britain, the cultural formation of engineers arose from a tradition in which industry was expected to play a key part. The professionalization of engineering in Great Britain focused on traditional craftsmanship and apprenticeship rather than theoretical learning (Brosan, 1972; Pratt, 1997; Sjöstrand, 2013). In Ireland, technical education began under British influence as an applied type of study relevant to trade and industry (Walsh, 2018, pp.143–144). The first technical educational institution in Dublin, now the Technological University of Dublin, started as “the product of a coalition between the Corporation, business interests in the city and the newly formed Dublin Trades Council” (Walsh, 2018, p.130). Instruction had the role of preparing for employment in industry and trade in close liaison with employers’ associations. It included courses in commercial subjects such as accountancy and economy as well as apprenticeships (Duff et al., 2000; Walsh, 2018).

Outside Europe, the vision of engineering education rooted in the “shop culture” was predominantly found in the USA, where it developed under the influence of a professional body trained through apprenticeship and who portrayed engineering as a commercial occupation (Calvert, 1967; McMahon, 1984; Seely, 1999b). Although envisioned to mix elements of the science and market orientations, it was the latter orientation that prevailed in the US engineering education. Notable in this sense is the observation of Alois Riedler, who during his visit in 1894 noted that while American students have impressive laboratories, they “lacked sufficient training in maths or physics” (Gispen, 1989).

In its beginnings, Massachusetts Institute of Technology aimed to serve the interests of industry and commerce as much as focusing on basic research and the advancement of science (Angulo, 2009; Etzkowitz, 1988). To support this educational ideal, its founder, William Barton Rogers, introduced dedicated teaching methods, spaces and departments. Among these is a museum displaying American industrial and agricultural innovations for the purpose of higher learning and the Society of Arts, which was a department for “keeping abreast of recent science-related inventions, products, and processes, both domestic and foreign”, whose members would “recommend experiments with products, processes, and machinery worthy of further investigation” (Angulo, 2009, p.88, p.94). Another innovation was the centrality of “laboratory exercises” where faculty would “not only demonstrate experiments as part of lecture presentations, but also supervise student experiments with laboratory apparatus” (Angulo, 2009, p.95). Through these initiatives, Rogers hoped to attract students from industrial classes. Reflecting a stronger market-driven vision were the College of Engineering and Applied Science of the University of Cincinnati (Harwood, 2006, p.56) and Georgia Institute of Technology, with the latter defending in the late nineteenth century its practically oriented curriculum as an example of “practice against theory; the shop against the study; the hammer against the book” (Harwood, 2005, p.11).

In Colombia, the National School of Mines in Medellin cultivated the "longstanding position" according to which engineers "should serve as entrepreneurs and managers in [private] companies." As was the case with many European TUs, its location was a "commercial and industrial center that dominated the most important national export product of the time” (Valderrama et al., p.829).

The market orientation is manifest in contemporary engineering education through two types of discourse. First, there is the employability discourse, according to which “the focus of training must increasingly be on employability,” highlighting the “urgent need for a concerted effort […] to ensure that a well-trained flexible workforce is available as a means of sustaining a national competitive advantage in a world of mega-competition” (Richardson, 2000, p.179). This approach is fostered through professional skills courses, labor-market surveys of desired graduate skills or curricular content, and the creation of industry advisory boards within TUs (Case, 2014; Shekhawat, 2020; Martin, Bombaerts & Johri, 2021). The market orientation is the outcome of increasing calls for engineering education to be more responsive to the needs of industry (Downey & Lucena, 1995).

Second, there is the entrepreneurial discourse promoting the entrepreneurial university as “a centre of innovation and self-sufficiency in a turbulent age” (Clark, 1998). This approach bloomed with the signing in the USA in 1980 of the Bayh-Dole Act (Audretsch & Belitski, 2021), at a time when concerns about competitiveness elevated engineering to the status of “a national problem” (Downey & Lucena, 1995, p.183). The professional identity promoted is of the engineer as a hero entrepreneur (Byers et al., 2013). The template of entrepreneurial universities was shaped by engineering-based higher education institutions (Benner, 2020; Clark, 1998). The main traits are strong steering, a culture of entrepreneurial achievements, a commitment to financial expansion, agility, and openness to change (Gibb, 2013; Pinheiro & Stensaker, 2014). The approach is cultivated through research spinoffs, student entrepreneurial projects or dedicated university departments and learning spaces, such as technology-transfer offices or innovation spaces (Etzkowitz, 2003; Hülsbeck et al., 2013; Rasmussen et al., 2006). According to Benner (2020), young TUs such as Twente, Warwick, Joensuu and Strathclyde are representative of the entrepreneurial focus.

Some of the pedagogical approaches of the market orientation are reflected in the impactful movement CDIO-Conceive, Design, Implement, Operate new products, processes, and systems (Norrman & Hjelm, 2017, Edström, 2017b). According to Kristina Edström (2018), one of the CDIO founders, the movement emerged in the late 1990s with the aim of “improving an overly theoretical education by integrating also necessary professional aspects.” In the background, we find debates about the future of engineering education which “could be interpreted as reactions to the lack of professional preparation” (Edström, 2018, p.51). CDIO emphasizes design- and project-based learning (Crawley et al., 2014; Edström et al., 2020), and more currently has extended to accommodate challenge-based learning (Kohn Rådberg et al., 2020). The educational focus is on “learning the process through which an idea or invention is connected to useful application and a means of creating access to it (innovation) in a scalable way (entrepreneurship)” (Weilerstein & Byers, 2016, p.2).

Overall, the current market orientation recognizes STEM knowledge as “a force of production in its own right” (Barnett & Guzmán-Valenzuela, 2021, p.4). This orientation is underpinned by a fundamental socio-economic change towards a knowledge-based society (Bassano et al., 2019), reflected in a curriculum framework that prioritizes economically powerful skills (Ward, 2012) and knowledge commercialization (Audretsch & Belitski, 2021). Some proponents root the market orientation of engineering education in the “logic of the marketplace” (Slaughter & Rhoades, 2004) and neo-liberal beliefs about the positive effects of competition, efficiency and work productivity (Conlon, 2008; Pinheiro & Stensaker, 2014). In the market orientation, the creation of new knowledge and understandings is valued through its impact on the wider world, “preferably in the form of an economic return” (Barnett, 2012, p.36).

This approach is not without critics. It has been argued that an extreme focus on getting graduates to adapt to the needs of the labour market may lead to social myopia when ignoring problematic employment practices or social conditions (Conlon, 2008, p.154). Reflecting on the US context, Noble (1979) described engineering education as a system explicitly designed to serve corporate interests. The most fervent critics deplore the capturing of universities by “cognitive capitalism” (Boutang, 2012; Roggero, 2011) or “knowledge capitalism” (Peters, 2013), which leaves unchallenged the production and innovation paradigm (Vinsel & Russell, 2020). Innovation is considered both “as value-in-itself and as panacea” (Russell & Vinsel, 2019, p.250). As such, some blamed the market orientation for having contributed to the “privatisation of everything” (Burawoy, 2005, p.263), including vital resources like water (Petrella, 2001). According to its critics, market-driven engineering education neglects the commitment toward a just economic distribution (Johnston et al., 1996) and bridging the gap between what technology could provide for society and its actual contribution (Cooley, 1978).

### The Social Orientation

A more recent approach emphasizes engineers’ social role. At its core, the social orientation was reflected in the engineering curriculum through content purporting to ethics, citizenship, liberal arts, critical theory, global and cultural awareness, and Science and Technology Studies (STS).

The rise of the socially oriented TU has been slow and conducted in parallel to efforts of establishing the primacy of societal considerations and accountability of the engineering profession (Weil, 1984, p.343; Mitcham, 2009). It can be credited to two forces. One is represented by individual engineers pushing for change toward the primacy of public interest (Mitcham, 1994). The other is the force of citizen movements demanding accountability for the social and environmental impact of engineering developments, following the controversies related to the use of nuclear weapons during WW2 and several aviation and car safety failures (Weil, 1984; Mitcham, 1994; Seely, 2005).

Six key moments stand out in the emergence of the social orientation: (i) the publication of pedagogical interventions in the *Journal of Engineering Education* in 1960s (Wisnioski, 2009, p.761) (ii) the genre of technology and society emerging in the late 1960s linked with small publishing houses (Wisnioski, 2009, p.757), (iii) the 1968 survey *Liberal Learning for the Engineer* conducted by Olmsted on behalf of the American Society of Engineering Education, which prompted nearly two hundred technical colleges in the US to experiment with teaching interventions that addressed technology’s societal implications (Wisnioski, 2012, p.165); (iv) the report commissioned by the Hastings Center in 1977 mapping the status and prospects of engineering ethics in the US and addressing for the first time the aims and content of engineering ethics education, the qualifications required for instructors and the available teaching material (Baum, 1980; Mitcham & Englehardt, 2019); (v) the first textbooks on engineering ethics published in the late 1970s and 1980s (Mitcham, 2009; Weil, 1984) and (vi) the signing of the Washington Accord in 1989 by the accrediting bodies of the USA, Canada, UK, Ireland, Australia and New Zealand, which included among its graduate attributes “societal, health, safety, legal and cultural issues and the consequent responsibilities relevant to professional engineering practice” (International Engineering Alliance, 1989). The Accord is considered to have paved the way for the introduction of ethics as an accreditation criterion and in the engineering curriculum in signatory countries across the world from 2000 onwards (Lattuca et al., 2006).

The first dedicated courses and programs representative of the social orientation flourished at elite universities, such as Harvard or CalTech, and in a small group of liberal arts colleges. An example is Dartmouth Engineering, whose faculty were drawn to theories of technological politics (Wisnioski, 2012). Under the administration of Frank Warren Garran, Darthmouth sees in 1942 the launch of the Tuck-Thayer program, consisting of three years of liberal arts topped with two years of engineering and business (Thayer School of Engineering, n.d.). This vision of engineering education promoting breadth over depth through liberal arts was further pursued in the 1960s through several measures implemented by Dean Myron Tribus, including the abolishment of disciplinary departments (Seely, 2010, p.94).

If the technical and market-driven approaches made their way from Europe to the USA via engineers educated at European institutions (Karvar, 1995)^4^, the expansion of the socially oriented TU originates in the USA and only later became manifest in other parts of the world. Among the TUs in Europe, Delft has developed for over two decades engineering ethics courses emphasizing responsible research and innovation, design for values, and risk ethics (Taebi & Kastenberg, 2019; van Grunsven et al., 2021).

In its inception, the socially oriented TU fostered various curricular approaches, ranging from humanizing engineering via liberal education (Bucciarelli & Drew, 2018), teaching systems analysis to produce professional socio-technologists, introducing non-engineers to technological thinking or creating social-scientific experts outside of engineering (Wisnioski, 2012). More recently, we witness the emergence of non-mainstream currents promoting global and cultural awareness (Downey et al., 2006; Johri & Jesiek, 2014; Luegenbiehl & Clancy, 2017; Martin et al., 2023), social justice (Baillie, 2020; Leydens & Lucena, 2017; Niles et al., 2020), activist engineering (Karwat, 2020), feminism (Riley, 2013), decolonial movements (Cordeiro Cruz, 2021; Kutay et al., 2018; Lord et al., 2019; Seniuk Cicek et al., 2021), liberative approaches (Bowen & Johnson, 2020; Riley, 2003), critical participation (York, 2018) and community participation in technology development and assessment (Kaplan et al., 2021). Through these pedagogical approaches, the social orientation strives to develop the identity of engineering graduates as citizens and community members.

Despite promoting a vision of societal service, this orientation is criticized for its potentially limited focus on altering the contextual aspects preventing ethical behaviour. The social orientation may not fully account for technology’s role in perpetuating power structures and methods of resistance in engineering practice (Jones, 2021; Bucher, 2018). It presupposes a bottom-up approach towards societal change, yet the military origins of engineering imposed top-down hierarchical processes that reinforce the status quo of current power relations over the public good (McGowan & Bell, 2020). For example, case studies have a diminished focus on power relations, ensuring equity, empowering individuals, fostering a dissenting voice, criticizing the profession and professional institutions, or addressing the “structural issues affecting an engineer’s agency” (Martin et al., 2021b, p.55; Rottmann & Reeve, 2020; Morrison, 2020; Lawlor, 2021). Following Huckle (2017, p.70), it can be argued that the social oriented engineering education can do better in conveying “powerful knowledge” that would expose students to reflection on “the structures and processes at work in the world that lead to injustice, a lack of democracy, and a failure to realise sustainable forms of development,” as well as the “ideology that masks these structures and processes.” Examples include addressing the culture of oppression against people of colour rooted in algorithm bias (Umoja Noble, 2018) or the impact of surveillance technology on civil liberties (Lyon, 2007). The social orientation is also in its infancy stage when it comes to reflecting on how engineering education replicates Western cultural paradigms or caters to specific ethnic or gender groups to the exclusion of women, people of colour or minorities (Lord et al., 2019).

### The Political Orientation

The political orientation of engineering education is linked to a major role engineers had in national plans for military operations and defence during the eighteenth and nineteenth centuries (Singh, 2012). As nations focused on territorial unification, there was a need for specialised professionals able to contribute to increasingly technical domains purporting to communication, roads, canals and shipbuilding, armaments and fortification (Karvar, 1995). On the European continent, shortages in expertise led France, Germany and the UK to pioneer the development of formal education for engineers (Karvar, 1995; Bucciarelli et al., 2009). The engineering corps proved essential during the Napoleonic Wars, but also in the US Independence War (Lienhard, 1998). In Britain, in 1812, the experience of the Peninsular War revealed the importance of fortifications, leading to the establishment of a Royal Engineering School at Chatham (Bucciarelli et al., 2009, p.2). Other examples offered by Karvar (1995, pp.85–86) include the Virginia Military Institute, where students were part of the state militia during their enrolment. In Saint Petersburg, engineers had the same status as army officers, while in Japan, the building of a shipyard was accompanied by the creation in 1867 of a technical school which included a track restricted to Samurai for training shipbuilding engineers. Thus, the political orientation saw the engineers’ role overlapping with that of a soldier in state’s service.

In the twentieth century, we also encounter a notable role of engineers as policymakers. A strong example is the collaboration between the US President Franklin D. Roosevelt and his scientific advisor, the engineer Vannevar Bush (1945), which led to the historical document *Science, the Endless Frontier.* MIT and Harvey Mudd College initiated two pioneering programmes reflecting the political role of engineering. In 1947, under the direction of Warren K. Lewis, MIT undertook a two-year survey focused on developing world leaders (Wisnioski, 2012, p.178). Jerome Wiesner, who joined MIT in the 1966 first as dean of science and later became president, further advocated for a “new breed of socially and politically savvy technologists” and envisioned the institution as “an incubator of technical leaders who would serve civil society with results” (Wisnioski, 2012, pp.180–181). This ideal culminated in 1975 with the official introduction of a program in *Technology Studies*, with courses such as “Theories of Technological Society and Politics” and having as members the political scientist Langdon Winner, the historian Charles Weiner, and the engineers Irving Kaplan and Louis Bucciarelli (Wisnioski, 2012, p.182). At Harvey Mudd College, the program *Quest for Commonwealth* was introduced in the 1970s by Theodore Waldman, whose goal was to instil the “understanding that the political legal and social problems that men had dealt with was as important as suggesting a solution to them” (Wisnioski, 2012, p.177). The program included reading Thucydides’ *History of the Peloponnesian War* and reflections on how legislative bodies deliberate over public issues (Wisnioski, 2012, p.177).

More recently, the policy role of engineers is closely linked with the technologization of society and automation of work. Mitcham (2009) has identified a “policy turn” in engineering education, concerned with transforming institutional arrangements and policy directives affecting engineering practice, but also with structural issues such as overconsumption (Swearengen & Woodhouse, 2003). It was argued that to enhance engineers’ agency towards responsible practice, it is crucial to first develop the means to change the context of practice through laws, policies or regulations, and to strengthen support structures, such as professional associations or civic groups (Son, 2008; Zandvoort, 2005). Beder (1998, pp.175–176) agrees that laws imposing “previously non-existent constraints” can become “inducement mechanisms” for technological innovations protecting the environment. Given the need for policy modifications, this orientation promotes the curricular adoption of change-agent skills and opportunities to take action on and off campus (Rowe, 2007, p.324).

The aims of the political and social orientations are similar in their engagement with societal challenges and contribution to the public good. But while the social orientation emphasizes this engagement through bottom-up change measures, the political orientation is focused on enabling engineers to engage competently with the elite and change the world from top-to-bottom, through laws, regulations and political decision-making.

Common curricular approaches include a focus on how public policy and patterns of regulation can lead to more sustainable outcomes (Donnelly & Boyle, 2006, p.1), knowledge about national and international standards, directives, regulations, and legislation, product liability, contract documents and planning requirements, security, privacy and GDPR, intellectual property and patent laws and simulations of policy-making processes (Martin et al, 2020; Bekkers & Bombaerts, 2017; Conlon & Zandvoort, 2011).

Nevertheless, the political orientation finds itself at the periphery of engineering education (Morgan et al., 2020). It is also considered to insufficiently question the paradigms of innovation and economic growth that are core to engineering practice (O’Neill, 2012). TUs predominantly situate their teaching within mainstream economic currents that envision unlimited economic growth as possible and desirable. The discussion of alternate ways of conceiving economic activity or promoting the reuse and maintenance of existing technological artefacts is largely absent from engineering education (Johnson & Siller, 2021; Russell & Vinsel, 2019). These might include making room in the curriculum for lecture topics, reflective assignments or design challenges linked to degrowth, agrowth or bioeconomics practices and theories (Kallis & Schneider, 2008). The political engineering education could complement its current teaching through knowledge of alternative policies oriented at sustainable production, promoting refurbishment, or designing economic systems for the people and planet (Kopnina, 2018, p.21; Jackson & Senker, 2011).

### The Ecological Orientation

The ecological orientation is the most recent orientation of TUs. It gained attention with the increasing calls for engineers to address the three pillars of the sustainable development goals (SDG), tackle climate change, and develop cradle-to-cradle strategies. This orientation acknowledges that TUs’ role is broader than knowledge generation and transfer, comprising contributions to a more sustainable and just future (Cortese, 2003; Giesenbauer & Müller-Christ, 2020) and the development of a circular economy (Kirchherr & Piscicelli, 2019). According to this orientation, TUs need to address on a regional or a global level the minimization of negative environmental effects generated in the use of their resources, via teaching, research, outreach, partnership, and stewardship (Velazquez et al., 2006).

The curricular focus is on the needs of present and future generations and the transition to sustainable societal patterns (Segalàs, 2009). The driver for formally considering environmental, ecological and sustainable aspects in education was the Stockholm Conference in 1972, which led to several academic declarations, charters and partnerships (Lozano et al., 2013, p.12), and the report *Our Common Future* of the UN Brundtland Commission (WCED, 1991). At the same time, global justice issues were acknowledged in the 1970s via counter-culture movements concerned with the Gandhian ideal of technology appropriation (Nieusma & Riley, 2010). More recently, at the intersection of both areas of concern and seeking to incorporate all three pillars of sustainable development, we find the emergence of global energy justice proponents and movements (Bombaerts et al., 2020; Sovacool & Dworkin, 2014).

Among the first TUs that implemented ecological curricular components are Monterrey Tec, Delft University of Technology (Lozano, 2006), Royal Melbourne Institute of Technology (Holgaard et al., 2016), Chalmers University of Technology, the Polytechnic University of Catalonia (Holmberg et al., 2008), The Tokyo Institute of Technology and Colorado School of Mines (Mitcham & Munoz, 2010; Wisnioski, 2012).

The practices associated with this orientation relate to adaptive management to emerging topics (Brown, 2012), cooperation with NGOs (Perez-Foguet et al., 2018)^5^, initiatives for greening the whole campus (Leal Filho et al., 2018), interdisciplinarity and silo-breaking (Blanco-Portela et al., 2017), as well as the creation of new departments such as green offices (Bautista-Puig & Sanz-Casado, 2021) or dedicated programs in Environmental or Humanitarian engineering (Mitcham & Munoz, 2010). Teaching is focused on learning by doing and doing by learning (Loorbach, 2007), whole systems thinking (Sterling, 2005; Loorbach & Rotmans, 2006, p.10), closed-loop systems (Kopnina, 2021), biomimicry tools and methods (Martínez-Acosta et al., 2023; Stevens et al., 2021), problem reframing (Sterling & Schumacher Society, 2001), technology appropriation and critiques of global technology transfers (Nieusma & Riley, 2010), questioning dominant paradigms and privilege in international development (Xavier et al., 2019), humanitarian engineering (Lucena et al., 2007; Mazzurco & Murzi, 2017), cross-cultural design projects (Fairfax & Lee, 2016) or eco-designs (Nickel et al., 2022). The identity promoted through these practices is of the engineer as a planetary citizen (Thompson, 2001), transition manager (Loorbach, 2007), or change agent (Van Poeck et al., 2017).

Nonetheless, there is a risk that the explicit focus on sustainability in the public mission and value statements of universities turns into greenwashing (Sonetti et al., 2016). Greenwashing is defined as the mismatch between an institution’s poor environmental performance and positive communication about environmental performance (Delmas & Burbano, 2011). This is “a deliberate act” to either hide potentially harmful environmental practices or to falsely portray the university’s practices as eco-friendly (Mitchell & Ramey, 2011). Given that the green market is proliferating (Delmas & Burbano, 2011) and students are increasingly attracted to degrees in sustainable engineering (SOS, 2021), TUs recognize the market value of green branding. At its worst, the ecological orientation is a shopwindow tied to neoliberalism that undermines its core values (Beveridge et al., 2014). In this sense, greenwashing is a strategic decision for TUs to make unwarranted or exaggerated claims of environmental friendliness in an attempt to gain market share (Dahl, 2010, p.247). Furthermore, this approach tends to emphasize the environmental pillar to the neglect of the socio-economic pillars of SDG (Jamison, 2013; Schank & Rieckmann, 2019). As such, the link to the social orientation is often overlooked as the dominant discourse on sustainability is cast in technical terms.

## Articulating the Responsibility of Technological Universities

As engineering education changed and broadened over time, and this process is ongoing, the responsibility of contemporary TUs is far from clear. Current societal challenges demand a broader role of TUs, which sees them as key contributors to the development and resilience of societies (Geschwind et al., 2019). This role is highlighted in new initiatives, such as the University Social Responsibility Network (Shek et al., 2017). Moreso, TUs are ideally placed to respond to “the skills-wisdom gap”, by which Martin (2006) draws attention to the increasing danger that technological expertise will surpass the ability of governments and people to direct it to constructive ends. Following reflections on the responsibilities that institutions have (Erskine, 2003), we extend this discussion to TUs and postulate that responsibility needs to be a component of the identity and activity of TUs.

Nevertheless, it is far from clear how to elaborate the responsibility of TUs. Furthermore, there is a disconnect between the wide range of historical orientations displayed by TUs and the homogenous understanding of responsibility proclaimed. Typically, it is the social responsibility that is made explicit and emphasized, either in mission statements or public discourse (Amorim et al., 2017). Yet it is unclear what the concept of responsibility refers to, nor does the concept provide a sufficient lens to grasp the distinct identities TUs may have. Given the gap between the homogenous formulation of societal responsibility and the heterogeneous orientations of TUs, it is crucial to articulate the responsibility of TUs, mindful of their different orientations.

In what follows, we address the second research question of our contribution, by enquiring what are the responsibilities of contemporary TUs? For this, we build on the historical review presented in the previous section to articulate five distinct responsibilities rooted in the different characteristics and orientations recorded in the history of engineering education. As such, to the prevailing suggestion that the locus of fostering responsibility lies with the social orientation of TUs, we respond that a focus on responsibility can permeate all orientations instantiated in the history of engineering education. Each orientation can be seen as a vehicle for promoting and fostering the development of engineering responsibility. Nevertheless, each orientation can be linked with specific understandings of responsibility, as rendered in Table 1:

- *Scientific responsibility* consists of the “appropriate application of scientific methods, the accurate reporting of results and open dissemination of findings” and “the consequences of research” (Rhodes & Sulton, 2010). It is associated with values pertaining to scientific discovery, research integrity, excellence, knowledge of disciplinary fundamentals, technical expertise, objectivity, open science and academic freedom.
- *Professional responsibility* follows professional conduct codes or targets corporate social responsibility when pursuing values purporting to product and process innovation, scalability, efficiency, effectiveness, competitiveness, profit and productivity.
- *Civic responsibility* prioritises people and communities as ends in themselves. It is linked with values such as justice, inclusiveness, care, equity, fairness, accessibility, subjectivity and empowerment.
- *Legal responsibility* focuses on the legal exercise of power, via top-down political decision-making or setting regulations or policies guiding technological design. Its corresponding values are legal accountability, public engagement and leadership.
- *Intra- and inter- generational responsibility:* considers moral obligations towards present and future generations. It acknowledges indirect reciprocity and ageless duties. It goes hand in hand with the principles and goals of sustainable development, as well as the values of universality and non-discrimination.

The five distinct articulations of responsibility permit the integration of the third mission of universities into all activities and practices of TUs, irrespective of their orientation (Karlsen et al., 2019). We thus call for contemporary TUs to become responsible institutions, by embracing in an explicit manner one or more conceptions of responsibility and aligning their vision with their practice. This extends to the collaborations pursued with different external actors in the co-creation of values (Table 1).

Stakeholders in value co-creation may include R&D centers and laboratories (science orientation), start-ups, SMEs and large industry companies (market orientation), NGOs, citizen associations, local communities, consumer and user groups (social orientation), local and national governments, think-tanks, national or international policy bodies (policy orientation), environmental associations, groups and networks, humanitarian bodies, underprivileged or at-risk communities, and communities affected by natural or man-made disasters (ecological orientation). A responsible TU needs to have criteria for selecting its external partners or funders that align with the responsibility it wishes to foster.

## Conclusion

The article started by noting that the TU is both an organization and an “assemblage of ideas” (Barnett, 2012, p.44). In its latter form, it allows for creative aspirations and conveys hope in the transformation of the former, as “a collective space to imagine new possibilities for the university’s agency” (Barnett, 2021, p.274). The contribution is twofold. First, it offers a historical overview of five major orientations that shaped engineering education. This allows university leaders and practitioners to reflect on the identity of their institutions, based on their pedagogical practices, conception of engineering and the role of the engineer fostered. Second, the paper puts forward five distinct ways for understanding the responsibility of contemporary TUs, complementing each orientation. The responsibility of TUs comprises scientific, professional, civic, legal, or intra- and inter-generational responsibilities.

As such, the paper serves as an invitation for TUs to use the lens rendered in Table 1 to engage in reflection about the orientations they display (their identity) and the change strategies towards the orientations they strive for (their agency). As Rover (2008, p.389) notes, “the key to change is first understanding what we are, and then taking steps toward what we are capable of becoming.” For the self-assessment of TU’s identity, we encourage the use of varied research techniques applied internally, via departmental peer-to-peer meetings, community events, or course questionnaires, as well as expert periodic curricular reviews conducted in consultation with stakeholders representing the different engineering roles highlighted in Table 1.

Considering the TU’s agency, the article calls for contemporary TUs to become responsible institutions, by making explicit in their practices the concept of responsibility arising from their orientation, by expanding the range of responsibilities they promote through the inclusion of teaching approaches linked with distinct orientation(s) and by having value-based criteria in place for establishing collaborations with external partners and funders in alignment with their proclaimed responsibility.

Our contribution also strives to open further empirical research into the prospects of developing responsible TUs, to complement our call and theoretical analysis of the responsibility of TUs grounded in the history of engineering education. An emerging research question is about identifying how TUs prioritize and implement the different range of responsibilities identified in our review study, and which stakeholders are involved in such processes. This implies understanding how TUs engage with internal and external stakeholders, including students, local communities, industry partners, government bodies, and nongovernmental organizations, to fulfil each of the distinct types of responsibilities. Additionally, it is important to acknowledge how TUs and external partners may influence each other's understanding of responsibility through their interaction. The impact of a responsible TU may extend to its entire ecosystem, prompting us to call for an examination of forms of education and learning that target multiple actors. At the same time, we should not omit to explore the key challenges and barriers that TUs face in fulfilling their responsibilities and the strategies employed for addressing them. This includes questions of how technological universities can be organized to expand their dominant orientation and develop strategies for hybridization, whereby a broader range of responsibilities are pursued.

Another future research area targets the development of instruments and tools for measuring and reporting the impact of activities pertaining to teaching, administration, curriculum, or campus design toward promoting and developing each type of responsibility. Furthermore, given the interconnected nature of current societal processes, it is important to enquire how TUs can collaborate with other stakeholders, including other universities via inter and intra-university alliances, to enact their responsibility missions. Such exchanges may bring about novel orientations in the TUs’ future, as well as new understandings of the responsibility of TUs that should not be omitted from future inquiries.

Ultimately, articulating a broad range of responsibilities that reflects the distinct historical orientations, allows us to better understand how contemporary TUs can respond to the multidisciplinary and complex nature of current societal challenges.

## Footnotes

1 Such as institutes of technology, polytechnical universities or engineering colleges.

2 We are aware of the limitations of our approach, which does not strive to enquire deeper, à la Gilbert Ryle, what is a university and where can the University of Oxford be found. Instead of tracing the metaphysical characteristics of universities in relation to their parts or encompassing structures, for simplicity purposes, we focus on ways in which conceptual models of universities have been traced based on their instantiated function, that might be insensitive to variations in the different metaphysical understandings of the concept of “university”.

3 Throughout the article, we are referring to the institutions by their current name, unless otherwise specified.

4 In his study, Karvar (1995) mentions the contribution of French Polytechnicians to the establishment of engineering schools, at the request of foreign governments, as well as in bringing their expertise to the creation of public works in other countries. Among these works are “the fortification of the Eastern coast of America by S. Bernard, the construction of the trans-Appalachian railroad by C. Crozet, the reconstruction of Saint Isaac's Cathedral in Saint Petersburg by G. Lame and E. Clapeyron, and the dam-building sites in Egypt led by French Saint-Simonians”.

5 Notable are the educational efforts of organisations or platforms such as Engineers Without Borders, Engineers Against Poverty, Engineering for Change, Engineers for Overseas Development and Engineers for a Sustainable World (Nieusma & Riley, 2010; Smith, Tran & Compston, 2020).

## References

[CR1] Ahlström G (1982). Engineers and industrial growth: Higher technical education and the engineering profession during the nineteenth and early twentieth centuries.

[CR2] Amorim, J. P. et al. (2017). *Guidelines for universities engaging in social responsibility*. UNIBILITY project. https://www.iau-hesd.net/sites/default/files/documents/io8_guidelines_final_version_2017-09-12_print.pdf

[CR3] Angulo AJ (2009). William Barton Rogers and the Idea of MIT.

[CR4] Angulo AJ (2012). The polytechnic comes to America: How French approaches to science instruction influenced mid-nineteenth century American higher education. History of Science.

[CR5] Audretsch DB, Belitski M (2021). Three-ring entrepreneurial university: In search of a new business model. Studies in Higher Education.

[CR6] Augier M, March JG (2011). The roots, rituals and rhetorics of change.

[CR7] Baillie C, Michelfelder D (2020). Engineering and social justice. The Routledge handbook of the philosophy of engineering.

[CR9] Barnett R (2011). Being a university.

[CR10] Barnett R (2012). Imagining the university.

[CR11] Barnett R, Rider S, Peters MA, Hyvönen M, Besley T (2021). Realizing the world-class university: An ecological approach. World class university. Evaluating education normative systems and institutional practices.

[CR12] Barnett R, Guzmán-Valenzuela C (2022). The socially responsible European university: a challenging project. International Journal of Sustainability in Higher Education.

[CR13] Barry J (2012). The politics of actually existing unsustainability: Human flourishing in a climate-changed.

[CR505] Bassano, C., Carotenuto, A., Ferretti, M., Pietronudo, M.C., Coskun, H.E. (2019). Smart University for Sustainable Governance in Smart Local Service Systems. In: Ahram, T. (eds) Advances in Artificial Intelligence, Software and Systems Engineering. AHFE 2018. Advances in Intelligent Systems and Computing, 787. Springer, Cham. 10.1007/978-3-319-94229-2_20

[CR14] Baum RJ (1980). Ethics and engineering curricula.

[CR15] Bautista-Puig N, Sanz-Casado E (2021). Sustainability practices in Spanish higher education institutions: An overview of status and implementation. Journal of Cleaner Production.

[CR16] Beder S (1998). The new engineer: management and professional responsibility in a changing world.

[CR17] Bekkers R, Bombaerts G (2017). Introducing broad skills in higher engineering education: The patents and standards courses at Eindhoven University of Technology. Technology and Innovation.

[CR18] Benner M, Geschwind L, Broström A, Larsen K (2020). Are some technical universities better than others?. Technical universities. Higher education dynamics.

[CR19] Beveridge RM, McKenzie M, Bieler A, McNeil R (2014). Greenwashing in education: How neoliberalism and policy mobility may undermine environmental sustainability.

[CR20] Blanco-Portela N, Benayas J, Pertierra LR, Lozano R (2017). Towards the integration of sustainability in Higher Education Institutions: A review of drivers of and barriers to organisational change and their comparison against those found of companies. Journal of Cleaner Production.

[CR21] Bombaerts G, Jenkins K, Sanusi YA, Guoyu W, Bombaerts G, Jenkins K, Sanusi Y, Guoyu W (2020). Expanding ethics justice across borders: The role of global philosophy. Energy justice across borders.

[CR500] Borrego, M., Foster, M.J. and Froyd, J.E. (2014), Systematic Literature Reviews in Engineering Education and Other Developing Interdisciplinary Fields. *Journal of Engineering Education, 103*, 45–76. 10.1002/jee.20038

[CR22] Boutang YM (2012). Cognitive capitalism.

[CR23] Bowen CL, Johnson AW (2020). Critical and liberative theories: Applications in engineering education.

[CR24] Brosan GS (1972). The development of polytechnics in the United Kingdom. Paedagogica Europaea.

[CR25] Brown BC (2012). Leading complex change with post-conventional consciousness. Journal of Organizational Change Management.

[CR26] Bucciarelli L, Coyle E, McGrath D, Christensen H, Meganck M, Delahousse B (2009). Engineering Education in the US and the EU. Engineering in context.

[CR27] Bucciarelli L, Drew D, Kapranos P (2018). Breaking boundaries with liberal studies in engineering. The interdisciplinary future of engineering education: Breaking through boundaries in teaching and learning.

[CR28] Bucher T (2018). If…then: Algorithmic power and politics.

[CR29] Burawoy M (2005). The 2004 american sociological association presidential address: For public sociology. The British Journal of Sociology.

[CR30] Bush, V. & United States. (1945). *Science, the endless frontier: A report to the President*.

[CR31] Byers T, Seelig T, Sheppard S, Weilerstein P (2013). Entrepreneurship. Its role in engineering education.

[CR32] Calvert M (1967). The mechanical engineer in America, 1830–1910.

[CR33] Cape Peninsula University of Technology. (n.d.). *Vision and Mission.* https://www.cput.ac.za/about/vision

[CR34] Carberry AR, Baker DR, Dori Y, Mevarech Z, Baker D (2018). The impact of culture on engineering and engineering education. Cognition, metacognition, and culture in STEM education. Innovations in Science Education and Technology.

[CR35] Case JM, Young M, Muller J (2014). Problematizing curriculum: Contemporary debates in engineering education. Knowledge, expertise and the professions.

[CR36] Case JM (2017). The historical evolution of engineering degrees: Competing stakeholders, contestation over ideas, and coherence across national borders. European Journal of Engineering Education.

[CR37] Cech EA (2014). Embed social awareness in science curricula. Nature.

[CR38] Christensen H, Meganck M, Delahousse B, Christensen H, Meganck M, Delahousse B (2007). Introduction. Occupational Bildung in engineering education. Philosophy in engineering.

[CR39] Cincinnati University. (n.d.). College of Engineering and Applied Sciences*.* https://ceas.uc.edu/

[CR40] Clark BR (1998). Creating entrepreneurial universities: Organisational pathways of transformation.

[CR42] Colorado School of Mines. (n.d.). *Planning.* https://www.mines.edu/president/planning/

[CR43] Conlon E (2008). The new engineer: Between employability and social responsibility. European Journal of Engineering Education.

[CR44] Conlon E, Zandvoort H (2011). Broadening ethics teaching in engineering: Beyond the individualistic approach. Science and Engineering Ethics.

[CR45] Cooley M, Coates K (1978). Design, technology and production for social needs. The right to useful work.

[CR46] Cruz CC (2021). Brazilian grassroots engineering: A decolonial approach to engineering education. European Journal of Engineering Education.

[CR47] Cortese AD (2003). The critical role of higher education in creating a sustainable future. Planning for Higher Education.

[CR48] Crawley EF, Malmqvist J, Östlund S, Brodeur DR, Edström K (2014). Rethinking engineering education: The CDIO approach.

[CR49] Dahl R (2010). Greenwashing. Environmental Health Perspectives.

[CR50] Delmas MA, Burbano VC (2011). The Drivers of Greenwashing. California Management Review.

[CR51] Downey GL, Lucena JC, Jasanoff S, Markle GE, Peterson JC, Pinch T (1995). Engineering studies. Handbook of science and technology studies.

[CR52] Downey GL, Lucena JC, Moskal B, Bigley T, Hays C, Jesiek B, Kelly L, Lehr J, Miller J, Nichols-Belo A, Ruff S, Parkhurst R (2006). The globally competent engineer: Working effectively with people who define problems differently. Journal of Engineering Education.

[CR53] Duff T, Hegarty J, Hussey M (2000). The story of the Dublin Institute of Technology.

[CR54] Edström, K. (2017a). *Exploring the dual nature of engineering education: Opportunities and challenges in integrating the academic and professional aspects in the curriculum*. Doctoral thesis Stockholm, KTH Royal Institute of Technology, TRITA-ECE, 2.

[CR55] Edström K (2017). The role of CDIO in engineering education research: Combining usefulness and scholarliness. European Journal of Engineering Education.

[CR56] Edström K (2018). Academic and professional values in engineering education: Engaging with history to explore a persistent tension. Engineering Studies.

[CR57] Edström K, Geschwind L, Broström A, Larsen K (2020). Integrating the Academic and professional values in engineering education—Ideals and tensions. Technical universities. Higher education dynamics.

[CR58] Edström K, Malmqvist J, Roslöf J (2020). Scholarly development of engineering education—the CDIO approach. European Journal of Engineering Education.

[CR59] Erskine T (2003). Can institutions have responsibilities?.

[CR60] Etzkowitz H, Mendelsohn E, Smith MR, Weingart P (1988). The making of an entrepreneurial university: The traffic among MIT, industry, and the military, 1860–1960. Science, technology and the military. Sociology of the sciences (A yearbook).

[CR61] Etzkowitz H (2003). Research groups as ‘quasi-firms’: The invention of the entrepreneurial university. Research Policy.

[CR62] Fairfax D, Lee DY (2016). Cross-cultural design (CCD) learning reflective tool based on UK and Korea's collaborative design projects.

[CR63] Fox R, Guagnini A (2004). Education, technology and industrial performance in Europe 1850–1939.

[CR64] Flexner A (2002). Medical education in the United States and Canada. From the Carnegie Foundation for the Advancement of Teaching, Bulletin Number Four, 1910. Bulletin of the World Health Organization.

[CR66] Froyd, J. E. (2011). *Propagation and realization of educational innovations in the system of undergraduate STEM education*. White Paper Commissioned for the Characterizing the Impact of Diffusion of Engineering Education Innovations Forum.

[CR67] Geschwind L, Kekäle J, Pinheiro R, Sørensen MP, Sørensen MP, Geschwind L, Kekäle J, Pinheiro R (2019). Responsible universities in context. The responsible university.

[CR68] Geschwind L, Broström A, Geschwind L, Broström A, Larsen K (2020). Technical universities: A historical perspective. Technical universities. Higher education dynamics.

[CR69] Gibb AA (2013). Developing the entrepreneurial university of the future.

[CR70] Giesenbauer B, Müller-Christ G (2020). University 4.0: Promoting the transformation of higher education institutions toward sustainable development. Sustainability.

[CR71] Gispen K (1989). New profession, old order: Engineers and German society, 1815–1914.

[CR72] Graham R (2012). Achieving excellence in engineering education: Ingredients of successful change.

[CR73] Harwood J (2005). Technology’s dilemma: Agricultural colleges between science and practice in Germany, 1860–1934.

[CR74] Harwood J (2006). Engineering education between science and practice: Rethinking the historiography. History and Technology.

[CR75] Hashagen, U. *Der Mathematiker*, 280. In footnote 5, In Harwood, J. (2006). Engineering education between science and practice: Rethinking the historiography. *History and Technology*, 22(1), 53–79.

[CR76] Herkert JR (2001). Future directions in engineering ethics research: Microethics, macroethics and the role of professional societies. Science and Engineering Ethics.

[CR77] Heywood J (2005). Engineering education: Research and development in curriculum and instruction.

[CR78] Holgaard JE, Hadgraft R, Kolmos A, Guerra A (2016). Strategies for education for sustainable development—Danish and Australian perspectives. Journal of Cleaner Production.

[CR79] Holmberg J, Svanström M, Peet D-J, Mulder K, Ferrer-Balas D, Segalàs J (2008). Embedding sustainability in higher education through interaction with lecturers: Case studies from three European technical universities. European Journal of Engineering Education.

[CR80] Huckle J (2017). Powerful geographical knowledge is critical knowledge underpinned by critical realism. International Research in Gepgraphical and Environmental Education..

[CR81] Hülsbeck M, Lehmann EE, Starnecker A (2013). Performance of technology transfer offices in Germany. The Journal of Technology Transfer.

[CR82] International Engineering Alliance (1989). *The Washington Accord*. http://www.ieagreements.org/accords/washington/

[CR83] Jackson T, Senker P (2011). Prosperity without growth: Economics for a finite planet. Energy & Environment.

[CR84] Jamison A (2013). The making of green engineers.

[CR85] Jamison A, Kolmos A, Holgaard JE (2014). Hybrid learning: An integrative approach to engineering education. Journal of Engineering Education.

[CR86] Jessen E, Christensen SH, Meganck M, Delahousse B (2007). The romantic polytechnician in the philosophy and work of H.C. Ørsted. Philosophy in engineering.

[CR87] Johnson, G. R. & Siller, T. J. (2021). Engineering education for a zero growth economy. In *EESD2021: Proceedings of the 10th engineering education for sustainable development conference, building flourishing communities*, University College Cork, Ireland.

[CR88] Johnston SF, Lee A, McGregor H (1996). Engineering as captive discourse. Society for Philosophy and Technology.

[CR89] Johri A, Jesiek BK, Johri A, Olds BM (2014). Global and international issues in engineering education. cambridge handbook of engineering education research.

[CR90] Jones AH (2021). What is an educational good? Theorising education as degrowth. Journal of Philosophy of Education.

[CR91] Kallis, G. & Schneider, F. (2008). Well-being and ecological sustainability beyond growth. *d-GROWTH; collaborative project.* ICTA, Autonomous University of Barcelona.

[CR92] Kaplan LR, Farooque M, Sarewitz D, Tomblin D (2021). Designing participatory technology assessments: A reflexive method for advancing the public role in science policy decision-making. Technological Forecasting and Social Change.

[CR93] Karlsen J, Larrea M, Sørensen MP, Geschwind L, Kekäle J, Pinheiro R (2019). Does a responsible university need a third mission?. The responsible university.

[CR94] Karvar A (1995). Model reception in the domain of engineering education: Mediation and negotiation. History and Technology.

[CR95] Karwat DMA (2020). Self-reflection for activist engineering. Science and Engineering Ethics.

[CR96] Kircherr J, Piscicelli L (2019). Towards an education for the circular economy (ECE): Five teaching principles and a case study. Resources, Conservation and Recycling.

[CR97] Kohn Rådberg K, Lundqvist U, Malmqvist J, Hagvall Svensson O (2020). From CDIO to challenge-based learning experiences—Expanding student learning as well as societal impact?. European Journal of Engineering Education.

[CR98] Kopnina H (2018). Teaching circular economy: Overcoming the challenge of green-washing. Handbook of Engaged Sustainability.

[CR99] Kopnina H (2022). Exploring posthuman ethics: Opening new spaces for postqualitative inquiry within pedagogies of the circular economy. Australian Journal of Environmental Education.

[CR100] Kutay, C., Gunay, B., & Tobin, C. (2018). Cross-cultural construction engineering with Aboriginal communities. In *29th Australasian association for engineering education conference 2018,* 387–394. Engineers Australia.

[CR101] Larsen K, Geschwind L, Broström A, Geschwind L, Broström A, Larsen K (2020). Organisational identities, boundaries, and change processes of technical universities. Technical universities. Higher education dynamics.

[CR102] Larson MS, Beckert J, Zafirovski M (2006). Professions. International encyclopedia of economic sociology.

[CR103] Lattuca LR, Terenzini PT, Volkwein JF (2006). Engineering change: Findings from a study of the impact of EC2000.

[CR104] Lawlor R (2021). Teaching engineering ethics: A dissenting voice. Australasian Journal of Engineering Education.

[CR105] Leal Filho W, Raath S, Lazzarini B, Vargas VR, de Souza L, Anholon R, Quelhas OLG, Haddad R, Klavins M, Orlovic VL (2018). The role of transformation in learning and education for sustainability. Journal of Cleaner Production.

[CR106] Leboutte R (1995). From traditional know-how to technical skill. The process of training and of professionalization in the Belgian coalmining industry, 1700–1850. History and Technology.

[CR107] LeCuyer C (1992). The Making of a Science-based Technological University: Karl Compton, James Killian and the Reform of MIT. Historical Studies in the Physical Sciences.

[CR108] Leydens JA, Lucena JC (2017). Engineering justice: Transforming engineering education and practice.

[CR109] Lienhard, J. H. (1998). *The Polytechnic legacy*. Prepared for the ASME Management Training Workshop, August 22nd, 1998.

[CR110] Loorbach D, Rotmans J, Olshoorn X, Wieczorek AJ (2006). Managing transitions for sustainable development. Understanding industrial transformation—Views from different disciplines.

[CR111] Loorbach D (2007). Transition management: New mode of governance for sustainable development.

[CR112] Lord, S., Mejia, J. A., Hoople, G. D. & Chen, D. A. (2019). Special session: Starting a dialogue on decolonization in engineering education*. IEEE Frontiers in Education Conference*, (pp 1–3).

[CR113] Lozano R (2006). Incorporation and Institutionalization of SD into Universities: Breaking through barriers to change. Journal of Cleaner Production.

[CR114] Lozano R, Lukman R, Lozano FJ, Huisingh D, Lambrechts W (2013). Declarations for sustainability in higher education: Becoming better leaders, through addressing the university system. Journal of Cleaner Production.

[CR115] Lucena, J. C., Leydens, J., Mitcham, C., Munakata-Marr, J. & Straker, J. (2007). Humanitarian engineering: Ethics, theory, practices. *Ethics in Science and Engineering National Clearinghouse*, *356*.

[CR116] Luegenbiehl HC, Clancy RF (2017). Global engineering ethics.

[CR117] Lyon, D. (2007). *Surveillance studies: An overview*. Polity.

[CR118] Martin J (2006). The meaning of the 21st century: A vital blueprint for ensuring our future.

[CR600] Martin, D. A. (2020). Towards a Sociotechnical Reconfiguration of Engineering and an Education for Ethics: A Critical Realist Investigation into the Patterns of Education and Accreditation of Ethics in Engineering Programmes in Ireland. University of Dublin. 10.21427/7M6V-CC71

[CR602] Martin, D. A., Conlon, E., & Bowe, B. (2020). Integrating ethics across the engineering curriculum through sustainability and legislative topics. In J. van der Veen, N. van Hattum-Janssen, H-M. Järvinen, T. de Laet, & I. ten Dam (editors), SEFI 48th Annual Conference Engaging Engineering Education, Proceedings: Engaging Engineering Education (blz. 318-328). European Society for Engineering Education (SEFI). https://www.sefi.be/wp-content/uploads/2020/11/Proceedings-2020-web.pdf

[CR601] Martin, D. A., Bombaerts, G., & Johri, A. (2021). Ethics is a disempowered subject in the engineering curriculum. In H-U. Heiss, H-M. Jarvinen, A. Mayer, & A. Schulz (editors), Proceedings - SEFI 49th Annual Conference: Blended Learning in Engineering Education: challenging, enlightening – and lasting? 357-365. European Society for Engineering Education (SEFI). https://research.tue.nl/files/227713181/SEFI2021_Martin_Bombaerts_Johri_ETHICS_IS_A_DISEMPOWERED_SUBJECT.pdf

[CR119] Martin DA, Conlon E, Bowe B (2021). A multi-level review of engineering ethics education: Towards a socio-technical orientation of engineering education for ethics. Science and Engineering Ethics.

[CR120] Martin DA, Conlon E, Bowe B (2021). Using case studies in engineering ethics education: The case for immersive scenarios through stakeholder engagement and real-life data. Australasian Journal of Engineering Education.

[CR121] Martin DA, Polmear M, Hyldgaard Christensen S, Buch A, Conlon E, Didier C, Mitcham C, Murphy M (2023). The two cultures of engineering education: looking back and moving forward. Engineering, social science, and the humanities: Has their conversation come of age?.

[CR122] Martin DA, Gwynne-Evans A, Kazakova A, Zhu Q, Johri A (2023). Developing a global and culturally inclusive vision of engineering ethics education and research. International handbook of engineering education research.

[CR605] Martínez-Acosta, M., Vázquez-Villegas. P., Mejía-Manzano, L. A., Soto-Inzunza G. V., Ruiz-Aguilar K. M., Kuhn Cuellar, L., Caratozzolo, P. & Membrillo-Hernández, J. (2023). The implementation of SDG12 in and from higher education institutions: universities as laboratories for generating sustainable cities. Frontiers in Sustainable Cities 5:1158464. 10.3389/frsc.2023.1158464

[CR123] Massachusetts Institute of Technology – MIT. (2020). *A proposed values statement for MIT: Mind, hand, heart.* https://valuescommittee.mit.edu/

[CR124] Mazzurco, A. & Murzi, H. (2017). Evaluating humanitarian engineering education initiatives: A scoping review of literature. In Huda, N., Inglis, D., Tse, N., & Town, G. (Eds) *28th annual conference of the Australasian Association for Engineering Education (AAEE 2017).* Sydney: Australasian Association for Engineering Education, pp 415–422.

[CR125] McGowan VC, Bell P (2020). Engineering education as the development of critical sociotechnical literacy. Science & Education.

[CR126] McMahon AM (1984). The making of a profession: A century of electrical engineering in America.

[CR127] Meiksins P, Smith C (1996). Engineering labour: Technical workers in comparative perspective.

[CR128] Miller S (2019). Whither the university? Universities of technology and the problem of institutional purpose. Science and Engineering Ethics.

[CR129] Mitcham C, Shrader- Frechette K (1994). Engineering design research and social responsibility. Ethics of Scientific Research.

[CR130] Mitcham C (2009). A historico-ethical perspective on engineering education: From use and convenience to policy engagement. Engineering Studies.

[CR131] Mitcham C, Munoz D (2010). Humanitarian Engineering.

[CR132] Mitcham C, Englehardt EE (2019). Ethics across the curriculum: Prospects for broader (and deeper) teaching and learning in research and engineering ethics. Science and Engineering Ethics.

[CR133] Mitchell LD, Ramey WD (2011). Look how green i am! An individual-level explanation for greenwashing. Journal of Applied Business and Economics.

[CR134] Morgan DL, Davis KB, López N (2020). Engineering political fluency: Identifying tensions in the political identity development of engineering majors. Journal of Engineering Education.

[CR135] Morrison LA (2020). Situating moral agency: How postphenomenology can benefit engineering ethics. Science and Engineering Ethics.

[CR136] Nickel J, Duimering PR, Hurst A (2022). Distilling sustainable design concepts for engineering design educators. International Journal of Engineering Education.

[CR137] Nieusma D, Riley D (2010). Designs on development: Engineering, globalization, and social justice. Engineering Studies.

[CR138] Niles S, Contreras S, Roudbari S, Kaminsky J, Harrison JL (2020). Resisting and assisting engagement with public welfare in engineering education. Journal of Engineering Education.

[CR139] NMITE. (n.d.). *Disrupting education.* https://nmite.ac.uk/about/disrupting-education

[CR140] Noble DF (1979). America by design: Science, technology, and the rise of corporate capitalism.

[CR141] Norrman, C. & Hjelm, O. (2017). CDIO-based entrepreneurship courses as drivers of innovation in industrial segments. In *The 13th international CDIO conference, University of Calgary, Canada*.

[CR142] O’Neill DW (2012). Measuring progress in the degrowth transition to a steady state economy. Ecological Economics.

[CR143] Perez-Foguet A, Lazzarini B, Gine R, Velo E, Boni A, Sierra-Castaner M, Zolezzi G, Trimingham R (2018). Promoting sustainable human development in engineering: Assessment of online courses within continuing professional development strategies. Journal of Cleaner Production.

[CR144] Peters MA (2013). Education, science and knowledge capitalism: Creativity and the promise of openness.

[CR145] Petrella R (2001). The water manifesto: Arguments for a world water contract.

[CR146] Petrina S (2003). Two cultures of technical courses and discourses: The case of computer aided design. International Journal of Technology and Design Education.

[CR147] Pinheiro R, Stensaker B (2014). Designing the entrepreneurial university: The interpretation of a global idea. Public Organization Review.

[CR148] Pratt J (1997). The polytechnic experiment, 1965–1992.

[CR149] Rasmussen E, Moen Ø, Gulbrandsen M (2006). Initiatives to promote commercialization of university knowledge. Technovation.

[CR150] Rhodes C, Sulston J (2010). Scientific responsibility and development. The European Journal of Development Research.

[CR151] Richardson P (2000). Employability in a globalising economy: Implications for engineering training. Engineering Science and Education Journal.

[CR152] Riley, D. (2003). Pedagogies of liberation in an engineering thermodynamics class. In *American Society for Engineering Education annual conference*.

[CR153] Riley D (2013). Hidden in plain view: Feminists doing engineering ethics, engineers doing feminist ethics. Science and Engineering Ethics.

[CR154] Roggero G (2011). The production of living knowledge: The crisis of the university and the transformation of labour in Europe and North America.

[CR155] Rottmann C, Reeve D (2020). Equity as rebar: Bridging the micro/macro divide in engineering ethics education. Canadian Journal of Science, Mathematics and Technical Education..

[CR156] Rover DT (2008). Engineering identity. Journal of Engineering Education.

[CR157] Rowe DT (2007). Education for a sustainable future. Science.

[CR158] Russell AL, Vinsel L, Wisnioski M, Hintz ES, Kleine MS (2019). Make maintainers: Engineering education and an ethics of care. Does America need more innovators.

[CR159] Schank C, Rieckmann M (2019). Socio-economically substantiated education for sustainable development: Development of competencies and value orientations between individual responsibility and structural transformation. Journal of Education for Sustainable Development.

[CR160] Seely BE (1984). The scientific mystique in engineering: Highway research at the bureau of public roads, 1918–1940. Technology and Culture.

[CR161] Seely BE (1999). The ‘imbalance’ of theory and practice in American engineering education: Reforms and changes, 1920–1980. Icon.

[CR162] Seely BE (1999). The Other re-engineering of engineering education, 1900–1965. Journal of Engineering Education.

[CR163] Seely, B. E. (2005). Patterns in the history of engineering education reform: A brief essay. In *Educating the engineer of 2020: Adapting engineering education to the new century*, pp. 114–30. National Academies Press.

[CR164] Seely BE (2010). Review of Knowledge with know-how: Thayer school of engineering at Dartmouth, by E. Frye. History of Education Quarterly.

[CR165] Segalàs*,* J. (2009). *Engineering education for a sustainable future*. Tesi doctoral, UPC, Càtedra Unesco de Sostenibilitat.

[CR166] Seidl D (2005). Organisational identity and self-transformation: An autopoietic perspective.

[CR167] Seniuk Cicek J, Steele A, Gauthier S, Adobea Mante A, Wolf P, Robinson M, Mattucci S (2021). Indigenizing engineering education in Canada: Critically considered. Teaching in Higher Education.

[CR168] Shek DTL, Yuen-Tsang AWK, Ng ECW, Shek D, Hollister R (2017). USR network: A platform to promote university social responsibility. University social responsibility and quality of life.

[CR169] Shekhawat S, Sangwan K, Herrmann C (2020). Enhancing employability skills of engineering graduates. Enhancing future skills and entrepreneurship. Sustainable production life cycle engineering and management.

[CR170] Singh A (2012). Engineering mixes with politics. Construction Innovation.

[CR171] Sjöstrand G (2013). The field of technology in Sweden: The historical take-off of the engineering professions. Professions and Professionalism.

[CR172] Slaughter S, Rhoades G (2004). Academic capitalism and the new economy: Markets, state, and higher education.

[CR173] Smith J, Tran ANLH, Compston P (2020). Review of humanitarian action and development engineering education programmes. European Journal of Engineering Education.

[CR174] Son WC (2008). Philosophy of technology and micro-ethics in engineering. Science and Engineering Ethics.

[CR175] Sonetti G, Lombardi P, Chelleri L (2016). True green and sustainable university campuses? Toward a clusters approach. Sustainability.

[CR176] SOS – Students Organising for Sustainability International (2021). *Students, sustainability and education – Survey report*. https://sos.earth/survey-2020/

[CR177] Sovacool BK, Dworkin MH (2014). Global energy justice: Problems, principles, and practices.

[CR178] Sterling SR, Orr D (2001). Sustainable education: Re-visioning learning and change.

[CR179] Sterling, S. (2005). *Whole systems thinking as a basis for paradigm change in education: Explorations in the context of sustainability*. (PhD thesis), Centre for Research in Education and the Environment, University of Bath.

[CR180] Stevens L, de Vries M, Mulder K, Kopnina H, Kopnina H, Poldner K (2021). Biomimicry education as a vehicle for circular design. Circular economy: Challenges and opportunities for ethical and sustainable business.

[CR181] Stevens, R., Amos, D. L., Jocuns, A., & Garrison, L. (2007). Engineering as lifestyle and a meritocracy of difficulty: Two pervasive beliefs among engineering students and their possible effects. In *Proceedings of the 2007 American Society for Engineering Education annual conference and exposition*.

[CR182] Swearengen JC, Woodhouse E (2003). Overconsumption as an ethical challenge for engineering education. International Journal of Mechanical Engineering Education.

[CR183] Taebi B, Kastenberg WE (2019). Teaching engineering ethics to PhD students: A Berkeley-Delft initiative. Science and Engineering Ethics.

[CR184] Terrance S, Thompson DL (1991). The moral responsibilities of universities. Moral values and higher education.

[CR185] Thayer School of Engineering. (n.d.). *History*. Retrieved from https://engineering.dartmouth.edu/about/history

[CR186] Thompson J, Gleeson B, Low N (2001). Planetary citizenship: The definition and defence of an ideal. Governing for the environment.

[CR187] TU Eindhoven. (2018). *TUE strategy 2030: Drivers of change.* https://assets.tue.nl/fileadmin/content/Our_University/About%20our%20university/Publications/TUE_Strategie_2030-LR.pdf

[CR188] TU Munich. (n.d.). *Our mission statement.* https://www.tum.de/en/about-tum/our-university/mission-statement

[CR189] Umoja Noble S (2018). Algorithms of oppression.

[CR190] University College London. (n.d.). *History of UCL civil, environmental & geomatic engineering.* https://www.ucl.ac.uk/civil-environmental-geomatic-engineering/sites/civil_environmental_geomatic_engineering/files/prof_simons_history_copy.pdf

[CR191] Valderrama A, Camargo J, Mejía I, Mejía A, Lleras E, García A (2009). Engineering education and the identities of engineers in Colombia, 1887–1972. Technology and Culture.

[CR192] van den Hoven J (2019). Ethics and the UN sustainable development goals: The case for comprehensive engineering. Science and Engineering Ethics.

[CR193] van Grunsven, J., Marin, L., Stone, T., Roeser, S & Doorn, N. (2021). How to teach engineering ethics? A retrospective and prospective sketch of TU Delft’s approach to engineering ethics education. *Advances in Engineering Education.*

[CR194] Van Poeck K, Læssøe V, Block T (2017). An exploration of sustainability change agents as facilitators of nonformal learning: Mapping a moving and intertwined landscape. Ecology and Society.

[CR195] Velazquez L, Munguia N, Platt A, Taddei J (2006). Sustainable university: What can be the matter?. Journal of Cleaner Production.

[CR196] Vinsel, L., & Russell, A. L. (2020). *The innovation delusion: How our obsession with the new has disrupted the work that matters most*.

[CR197] York E (2018). Doing STS in STEM spaces: Experiments in critical participation. Engineering Studies.

[CR198] Xavier, P., Orbaek White, G., & Groves, C. (2019). Students exploring power and privilege in engineering design for sustainable community development. In J. Andrews, G. Knowles, R. Clark (Eds.), *The proceedings of the 7th annual conference of the UK and Ireland engineering education research network*, (pp 180–189).

[CR199] Walsh J (2018). Higher education in Ireland, 1922–2016: Politics, policy and power—A history of higher education in the Irish state.

[CR200] Ward SC (2012). Neoliberalism and the global restructuring of knowledge and education.

[CR201] World Commission on Environment and Development (WCED) (1991). Our common future.

[CR202] Weil V (1984). The rise of engineering ethics. Technology in Society.

[CR203] Weilerstein P, Byers T (2016). Guest editorial: Entrepreneurship and innovation in engineering education. Advances in Engineering Education.

[CR204] Wicklein RC (1997). Curriculum focus for technology education. Journal of Technology Education.

[CR205] Wisnioski MH (2009). “Liberal Education Has Failed”: reading like an engineer in 1960s America. Technology and Culture.

[CR206] Wisnioski MH (2012). Engineers for change: Competing visions of technology in 1960s America.

[CR207] Zandvoort H (2005). Good engineers need good laws. European Journal of Engineering Education.

